# Differences in risk factors for biochemical recurrence after radical prostatectomy stratified by the degree of obesity: Focused on surgical methods

**DOI:** 10.1038/s41598-020-67237-6

**Published:** 2020-06-23

**Authors:** Jungyo Suh, Sangjun Yoo, Juhyun Park, Min Chul Cho, Chang Wook Jeong, Ja Hyeon Ku, Cheol Kwak, Hyeon Hoe Kim, Hyeon Jeong

**Affiliations:** 1 Hospital Medicine Center, Department of Urology, Seoul National University Hospital, Seoul, South Korea; 2 Department of Urology, Seoul National University College of Medicine, Seoul, South Korea; 3 Department of Urology, Seoul Metropolitan Government-Seoul National University Boramae Medical Center, Seoul, South Korea; 4 Department of Urology, Asan Medical Center, Seoul, South Korea; 5 Department of Urology, Seoul National University College of Medicine, Seoul, South Korea

**Keywords:** Prostate, Surgical oncology

## Abstract

This study aims to evaluate differences in the risk factors for biochemical recurrence (BCR) for radical prostatectomy stratified by degree of obesity, focusing on the surgical method used. All 3099 patients who underwent radical prostatectomy in two medical centres from January 2008 to March 2018 were retrospectively reviewed. Patients were divided into three groups based on body mass index: ‘normal’, ‘overweight’, and ‘obese’. Risk factors associated with biochemical recurrence were compared between different degrees of obesity. We analysed the contributing risk factors of BCR-free survival using univariate and multivariable Cox hazard models. There were 378 (12.2%) and 1815 (58.6%) patients in the ‘obese’ and ‘overweight’ groups, respectively. Accordingly, 1324 patients underwent retropubic radical prostatectomy (RRP), and 1775 underwent robotic-assisted laparoscopic prostatectomy (RALP). Multivariable analysis showed that patients who underwent RALP had significantly lower BCR free survival in the ‘overweight’ and ‘obese’ groups than those who underwent RRP, with adjusted hazard ratios of 0.75 (95% CI 0.59–0.95, p-value = 0.01) and 0.55 (95% CI 0.33–0.90, p-value = 0.02), respectively. The degree of obesity was not directly associated with BCR-free survival. Robotic prostatectomy showed greater benefits in BCR-free survival than open prostatectomy in the ‘overweight’ and ‘obese’ groups.

## Introduction

There has been a remarkable increase in the prevalence of obesity in the last decade^1,2^. Obesity is not only related to overall health care problems but also linked to cancer development, cancer recurrence, and cancer-related mortality^3,4^. Although the contribution of obesity to the oncological outcomes of many cancers has been documented, its relationship with prostate cancer is still debated^4–9^. Limited evidence available from previous studies is mainly based on the relationship of obesity with many clinical factors that directly and indirectly affect clinical outcomes.

The clinical effect of obesity is much clearer with respect to surgical difficulty. Early research of the Shared Equal Access Regional Cancer Hospital (SEARCH) database found that obesity was related to positive surgical margins^10^. A more recent study has shown that obesity is related to worse functional outcomes and increased operation difficulty^11^. In cases of prostate cancer, robotic surgery has been developed to reduce the surgical difficulty and improve surgical outcomes by enhancing visualization and providing delicate manipulation. A recent meta-analysis revealed that robotic-assisted laparoscopic prostatectomy (RALP) showed superior results in functional outcomes^12,13^ than open retropubic radical prostatectomy (RRP), although there is still little evidence regarding oncological outcomes^14,15^.

We hypothesize that the degree of obesity has an effect on the oncological outcome in patients with prostate cancer due to the associated surgical difficulty in performing radical prostatectomy. The elevation of prostate specific antigen (PSA), which defined biochemical recurrence (BCR), is earliest event of local or distant recurrence after radical prostatectomy^16,17^. We designed a retrospective study to examine the clinical impact of the degree of obesity on BCR after radical prostatectomy. After adjusting for confounding factors, we assessed differences in the operation method affecting BCR stratified by the degree of obesity.

## Methods

### Ethical Approval

This study was approved by the Institutional Review Board of Seoul National University Hospital and Seoul Metropolitan Government-Seoul National University Boramae Medical Center. As this was a retrospective study with anonymization of data, the boards waived the requirement for informed consent from patients. All experiments were performed in accordance with relevant guidelines and regulations.

### Patient Selection and Definition

We retrospectively reviewed patients who had undergone radical prostatectomy from January 2008 to March 2018. The total number of patients was 3099, of whom 1324 had undergone RRP and 1775 had undergone RALP. We included patients who underwent radical prostatectomy for localized or locally advanced prostate cancer. The laparoscopic prostatectomy population was relatively small, about 135 patients, and was, therefore, excluded from the analysis. Patients who underwent adjuvant and neoadjuvant therapy were excluded from the analysis. Pre-treatment information (age, initial PSA levels, prostate volume, body mass index [BMI], and underlying diseases), operation related parameters (operation methods, pelvic lymph node dissection), pathologic parameters (pathologic Gleason grade group, pathologic T-stage, pathologic N stage, lymphovascular invasion, and positive surgical margin), and oncological outcomes (BCR-free survival) were reviewed.

BMI was calculated based on the height and weight of the patient measured 1 day before the operation. The degree of obesity was defined according to the BMI definition of the World Health Organization for the Asian population: ‘obese’ (≥27.5 kg/m^2^), ‘overweight’ (23–27.5 kg/m^2^), and ‘normal’ (<23 kg/m^2^)^18^. BCR was defined as having an elevated prostate-specific antigen (PSA) level above 0.2 ng/dL twice during the follow-up period^19^. The 5-year BCR-free survival was calculated for assessing the oncological outcome.

### Statistical Analysis

Continuous variables are summarized as mean ± standard deviation, and categorical variables are described as frequency (percentage). Continuous and categorical variables were compared using one-way analysis of variance and chi-square tests as appropriate in the three patient groups (‘normal’, ‘overweight’, and ‘obese’). The Kaplan-Meir curve and log-rank test were used to assess for BCR-free survival based on the operation methods and were stratified according to the degree of obesity. We also used the Cox-proportional hazard regression model to assess the potential contribution of clinical and pathologic parameters on BCR-free survival. After a literature review of pre-existing nomograms^20,21^, age, pre-operative PSA level, surgical margin, pathologic Gleason grade group, pathologic TN stage, and adverse pathologic features (lymphovascular invasion) were selected as potential risk factors for BCR. The degree of obesity and surgical methods, which are the factors of interest in this study, were also included in the regression model. All statistical analyses were performed using the SPSS package version 22.0 (IBM, Armonk, USA) and R package version 3.5.3 (www.r-project.org), and p values <0.05 were considered statistically significant.

## Results

### Patient Characteristics

The mean age of study patients was 71.4 ± 7.3 years, and the PSA level was 11.4 ± 14.7 ng/dL. Transrectal ultrasound was performed in 2321 patients (74.9%), and the average prostate volume was 41.6 ± 18.1 ml. The average BMI was 24.4 ± 2.8 kg/m^2^, and 450 (15.6%) patients had diabetes. Robotic prostatectomy was performed in 1775 patients (57.3%), and 1128 of these patients (36.4%) were diagnosed with pathologic T3 or greater. The numbers of patients in each Pathologic Gleason grade group (GGG) were 847 (27.3%), 1219 (29.7%), 718 (23.2%), and 315 (10.2%) for 1, 2, 3, and over 4. Pelvic lymph node dissection was performed in 874 patients (28.2%), and 138 patients (4.5%) had pathologic N1.

We divided the patients into three groups based on their BMI: 906 (29.2%), ‘normal’ group; 1815 (58.6%), ‘overweight’ group; and 378 (12.2%), ‘obese’ group. Comparison of the clinical characteristics and pathologic parameters between the study groups is described in Table 1. Statistically significant differences were seen among the clinical parameters of age, prostate volume, underlying disease, and selected operation methods. Factors directly associated with oncological outcomes (PSA level, pathologic stage, Gleason grade group, and pathologic features) were not statistically different among the three study groups.

**Table 1:** Clinical and pathologic characteristics of the three study groups, divided by body mass index (BMI).

Group	“Normal”	“Overweight”	“Obese”	p-value
	BMI < 23	BMI 23–27.5	BMI > 27.5	
	(N = 906)	(N = 1815)	(N = 378)	
Age (years)	72.2 ± 7.4	71.2 ± 7.2	70.6 ± 7.4	**<0.01**^**†**^
PSA (ng/dL)	12.1 ± 18.2	11.0 ± 13.5	11.2 ± 10.3	0.16^**†**^
Prostate Volume (cc)	39.1 ± 16.4	42.0 ± 17.8	46.1 ± 21.7	**<0.01**^**†**^
DM	106 (12.6%)	276 (16.3%)	68 (19.4%)	**0.01**^**‡**^
HTN	344 (38.0%)	925 (51.0%)	234 (61.9%)	**<0.01**^**‡**^
Operation methods				**<0.01**^**‡**^
RRP	436 (48.1%)	742 (40.9%)	146 (38.6%)	
RALP	470 (51.9%)	1073 (59.1%)	232 (61.4%)	
Gleason grade Group				0.36^**‡**^
1	251 (27.7%)	513 (28.3%)	83 (22.0%)	
2	352 (38.9%)	712 (39.2%)	155 (41.0%)	
3	207 (22.8%)	413 (22.8%)	98 (25.9%)	
4	55 (6.1%)	87 (4.8%)	22 (5.8%)	
5	41 (4.5%)	90 (5.0%)	20 (5.3%)	
Pathologic T stage				0.33^**‡**^
T2	569 (62.8%)	1172 (64.6%)	230 (60.8%)	
T3 or over	337 (37.2%)	643 (35.4%)	148 (39.2%)	
Pathologic N stage				0.31^**‡**^
N0	202 (22.3%)	445 (24.5%)	89 (23.5%)	
N1	47 (5.2%)	80 (4.4%)	11 (2.9%)	
Nx	657 (72.5%)	1290 (71.1%)	278 (73.5%)	
Surgical margin	291 (32.1%)	580 (32.0%)	140 (37.0%)	0.15^**‡**^
Apex margin	158 (17.4%)	333 (18.3%)	82 (21.7%)	0.19^**‡**^
Base margin	65 (7.2%)	157 (8.7%)	35 (9.3%)	0.32^**‡**^
Posterolateral margin	112 (12.4%)	203 (11.2%)	54 (14.3%)	0.21^**‡**^
Anterior margin	64 (7.1%)	144 (7.9%)	32 (8.5%)	0.62^**‡**^
Lymphovascular invasion	55 (6.1%)	123 (6.8%)	31 (8.2%)	0.38^**‡**^

^†^One-way ANOVA, ^‡^Chi-square test.

Regarding the operative methods, patients who underwent RALP were younger (70.1 ± 7.6 vs. 73.2 ± 6.5 years) and had lower PSA levels (10.2 ± 10.3 vs. 12.9 ± 19.0 ng/dL) than those who underwent RRP. BMI was higher in the RALP group; however, underlying hypertension was more prevalent in the RRP group. The positive surgical margin rate was not different between the RRP and RALP groups; however, the apex (16.2% vs. 21.6%, p-value < 0.01) and anterior margin positive rate (6.9% vs. 8.9%, p-value = 0.04) were significantly lower in the RALP group than in the RRP group (Table 2).

**Table 2:** Clinical and pathologic characteristics of retropubic radical prostatectomy and robot assisted laparoscopic prostatectomy.

Group	Open retropubic radical prostatectomy	Robot assisted laparoscopic prostatectomy	p-value
	(N = 1324)	(N = 1775)	
Age (years)	73.2 ± 6.5	70.1 ± 7.6	**<0.01**^**†**^
PSA (ng/dL)	12.9 ± 19.0	10.2 ± 10.3	**<0.01**^**†**^
BMI (kg/m^2^)	24.13 ± 2.8	24.6 ± 2.7	**<0.01**^**†**^
Prostate Volume (cc)	42.6 ± 19.0	40.9 ± 17.4	**0.02**^**†**^
DM	209 (17.0%)	241 (14.6%)	0.08
HTN	688 (52.0%)	815 (45.9%)	**<0.01**^**‡**^
Gleason grade Group			0.05^**‡**^
1	357 (27.0%)	490 (27.6%)	
2	493 (37.2%)	726 (40.9%)	
3	319 (24.1%)	399 (22.5%)	
4	84 (6.3%)	80 (4.5%)	
5	71 (5.4%)	80 (4.5%)	
Pathologic T stage			0.32^**‡**^
T2	829 (62.6%)	1142 (64.3%)	
T3 or over	335 (37.4%)	633 (35.7%)	
Pathologic N stage			**<0.01**^**‡**^
N0	310 (23.4%)	426 (24.0%)	
N1	88 (6.6%)	50 (2.8%)	
Nx	926 (69.9%)	1299 (73.2%)	
Surgical margin	422 (34.1%)	599 (31.5%)	0.12^**‡**^
Apex margin	286 (21.6%)	287 (16.2%)	**<0.01**^**‡**^
Base margin	113 (8.5%)	144 (8.1%)	0.67
Posterolateral margin	146 (11.0%)	223 (12.6%)	0.19
Anterior margin	118 (8.9%)	122 (6.9%)	**0.04**
Lymphovascular invasion	102 (7.7%)	107 (6.0%)	0.07^**‡**^

^†^One-way ANOVA, ^‡^Chi-square test.

**Figure 1:**
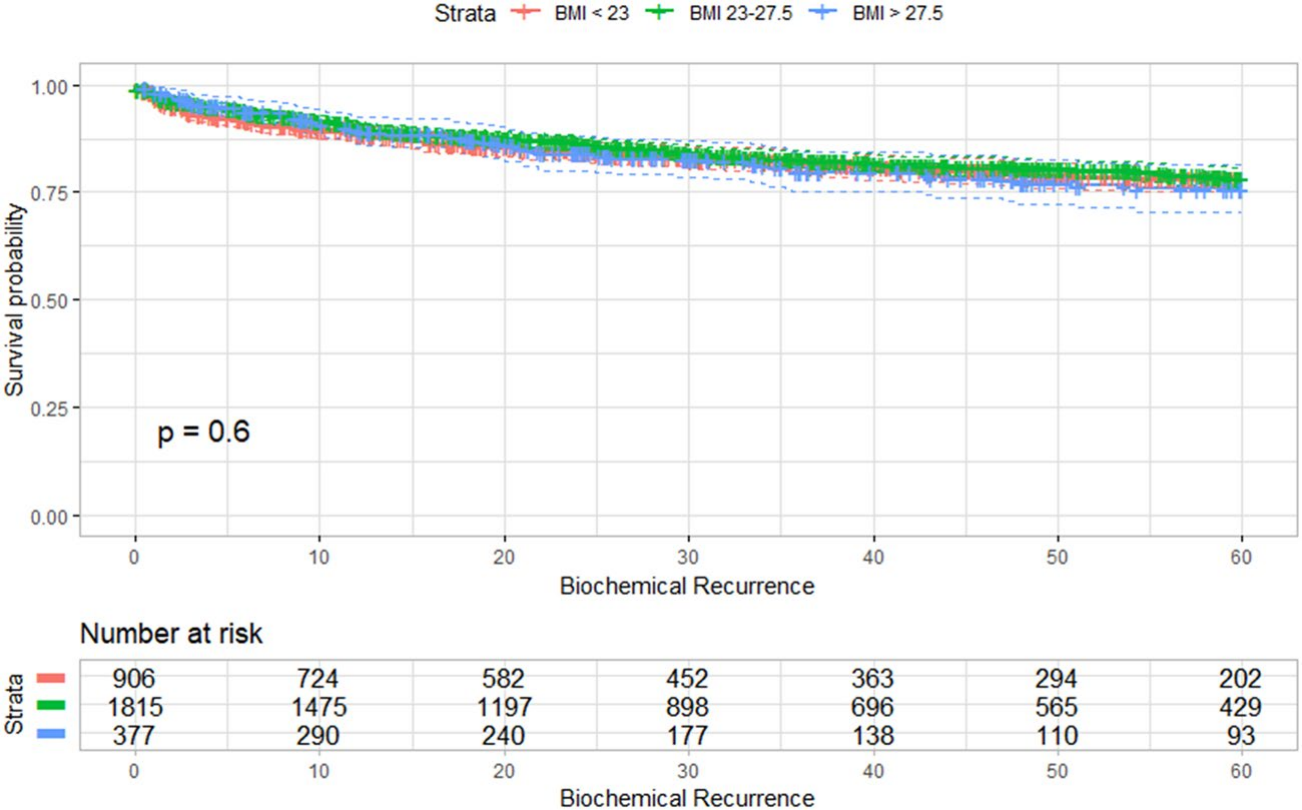
Kaplan-Meier curve of biochemical recurrence-free survival between the three obesity subgroups. There was no statistical difference between the degrees of obesity to biochemical recurrence free survival.

Upon using the Kaplan-Meier curve and log-rank tests, the degree of obesity was not found to be associated with BCR-free survival **(**Fig. 1). The univariate and multivariable analysis of predisposing clinical and pathologic factors with BCR-free survival in the study population is shown in Table 3. PSA level, surgical margin, Gleason grade group, pathologic T-stage, lymphovascular invasion, and surgical methods were the contributing factors for BCR. Regarding the surgical methods, RALP had significantly lower BCR (hazard ratio [HR] 0.82; 95% confidence interval [CI] = 0.68–0.97; p-value = 0.02) than RRP.

**Table 3:** Univariate and multivariable cox-proportional hazard analysis of clinicopathologic factors for biochemical recurrence-free survival in the entire study population.

	Univariate		Multivariable	
	HR (95% CI for HR)	p-value	HR (95% CI for HR)	p-value
Age (≥65 years)	1.11 (0.89–1.40)	0.35		
**PSA**				
<10	Reference		Reference	
10–20	2.71 (2.20–3.34)	<0.01	1.75 (1.41–2.17)	<0.01
>20	5.87 (4.75–7.25)	<0.01	2.26 (1.78–2.86)	<0.01
Operation method(RALP)	0.70 (0.59–0.83)	<0.01	0.82 (0.68–0.97)	0.02
Surgical margin(Positive)	3.04 (2.55–3.61)	<0.01	1.60 (1.32–1.95)	<0.01
**Gleason grade group**				
1	Reference		Reference	
2	3.45 (2.38–5.01)	<0.01	2.32 (1.59–3.39)	<0.01
3	9.82 (6.83–14.13)	<0.01	4.91 (3.36–7.20)	<0.01
4	14.23 (9.34–21.70)	<0.01	7.44 (4.80–11.53)	<0.01
5	19.72 (13.11–29.65)	<0.01	6.60 (4.25–10.24)	<0.01
Pathologic T stage(T3 or over)	4.1 (3.44–4.95)	<0.01	1.77 (1.44–2.19)	<0.01
**Pathologic N stage**				
N0	Reference		Reference	
N1	2.38 (1.81–3.12)	<0.01	1.25 (0.93–1.67)	0.14
NX	5.96 (4.59–7.74)	<0.01	0.85 (0.69–1.04)	0.13
Lymphovascular invasion (Positive)	4.35 (3.49–5.43)	<0.01	1.38 (1.07–1.77)	0.01
**BMI**				
BMI < 23	Reference			
BMI 23–27.5	0.93 (0.77–1.13)	0.45		
BMI > 27.5	1.04 (0.78–1.38)	0.79		

**Figure 2:**
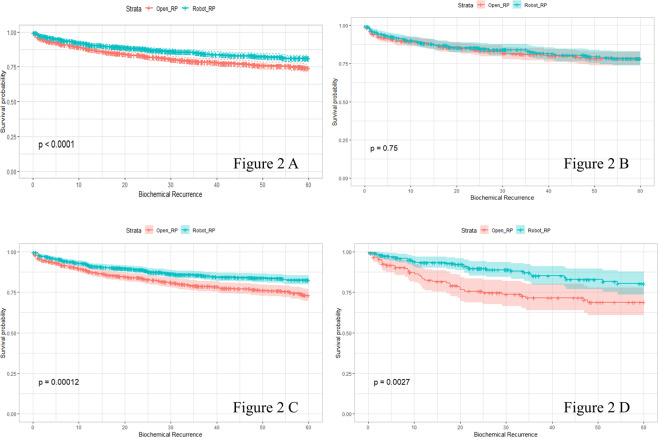
Kaplan-Meier curve of biochemical recurrence-free survival after open retropubic radical prostatectomy (RRP) and robot-assisted laparoscopic radical prostatectomy (RALP) in the entire study population (Fig. 1a). Subgroup analysis of the three groups of obesity is shown in Fig. 1b (“normal”, BMI < 23), Fig. 1c (“overweight”, 23 ≤ BMI ≤ 27.5) and Fig. 1d (“obese”, BMI > 27.5). P-values were calculated by the log-rank test and 95% confidence intervals are shown by the colored range around the survival curve. BMI, body mass index.

BCR-free survival was found to be 79.2% following RRP and 86.2% following RALP, as determined using the Kaplan-Meier analysis. For the entire study population, there was a significant association between BCR-free survival and the operation method (p-value < 0.01, Fig. 2A). After dividing the study population into three groups based on the degree of obesity, we performed Kaplan-Meier analysis for BCR-free survival following the surgical procedure. BCR-free survival was not significantly different between RRP and RALP in the ‘normal’ group (50.18 ± 1.00 vs. 50.79 ± 0.95, p-value = 0.75, Fig. 2B). BCR-free survival gradually decreased as the degree of obesity increased in patients who underwent RRP (50.18 ± 1.00, 49.42 ± 0.77, and 46.12 ± 1.94). In contrast, BCR-free survival gradually increased as the degree of obesity increased in patients who underwent RALP (50.79 ± 0.95, 52.75 ± 0.58, and 53.07 ± 1.21). Statistically significant difference in BCR-free survival between RRP and RALP was found only in the ‘overweight’ (49.42 ± 0.77 vs. 52.75 ± 0.58, p-value < 0.01, Fig. 2C) and ‘obese’ (46.12 ± 1.94 vs. 53.07 ± 1.21, p-value <0.01, Fig. 2D) groups.

Univariate and multivariable Cox-proportional hazard regression analyses for BCR-free survival were performed for each of the study groups (Table 4). In the ‘normal’ group, PSA, surgical margin, Gleason grade group, and pathologic T-stage were the contributing risk factors for BCR-free survival. The surgical method did not affect BCR-free survival in this subgroup. In the ‘overweight’ group (HR = 0.75, 95% CI = 0.59–0.95, p-value = 0.01) and ‘obese’ group (HR = 0.55, 95% CI = 0.33–0.90, p-value = 0.02), the surgical method was the contributing risk factor for BCR-free survival.

**Table 4:** Univariate and multivariable cox-proportional hazard analysis of clinicopathologic factors for biochemical recurrence free survival in three obesity sub-groups.

	“Normal”				“Overweight”				“Obese”			
	Univariate		Multivariable		Univariate		Multivariable		Univariate		Multivariable	
	HR (95% CI for HR)	p.value	HR (95% CI for HR)	p.value	HR (95% CI for HR)	p.value	HR (95% CI for HR)	p.value	HR (95% CI for HR)	p.value	HR (95% CI for HR)	p.value
Age (≥65 years)	1.05 (0.69–1.59)	0.83			1.18 (0.87–1.59)	0.28			0.99 (0.56–1.75)	0.96		
**PSA**												
<10	Reference		Reference		Reference		Reference		Reference		Reference	
10–20	3.79 (2.61–5.50)	<0.01	1.93 (1.30–2.86)	<0.01	2.52 (1.91–3.33)	<0.01	1.82 (1.37–2.41)	<0.01	1.51 (0.80–2.86)	0.20	1.15 (0.60–2.21)	0.68
>20	6.15 (4.12–9.19)	<0.01	1.92 (1.23–3.02)	<0.01	5.90 (4.46–7.81)	<0.01	2.56 (1.90–3.47)	<0.01	5.29 (3.07–9.13)	<0.01	2.35 (1.30–4.27)	<0.01
Operation method (RALP)	0.95 (0.67–1.23)	0.75			0.64 (0.51–0.81)	<0.01	0.75 (0.59–0.95)	0.01	0.49 (0.30–0.79)	0.03	0.55 (0.33–0.90)	0.02
Surgical margin (Positive)	3.71 (2.70–5.10)	<0.01	1.72 (1.21–2.46)	<0.01	2.85 (2.27–3.59)	<0.01	1.58 (1.22–2.03)	<0.01	2.49 (1.53–4.04)	<0.01	1.63 (0.95–2.78)	0.08
**Gleason grade group**												
1	Reference		Reference		Reference		Reference		Reference		Reference	
2	5.37 (2.43–11.88)	<0.01	3.22 (1.44–7.22)	<0.01	3.27 (2.02–5.28)	<0.01	2.30 (1.42–3.75)	<0.01	1.83 (0.73–4.62)	0.20	1.46 (0.57–3.76)	0.43
3	16.09 (7.36–35.14)	<0.01	6.85 (3.04–15.44)	<0.01	9.80 (6.16–15.60)	<0.01	5.34 (3.29–8.68)	<0.01	3.60 (1.44–8.98)	<0.01	2.52 (0.97–6.52)	0.06
4	18.14 (7.61–43.21)	<0.01	7.38 (2.97–18.34)	<0.01	13.17 (7.53–23.03)	<0.01	8.20 (4.63–14.53)	<0.01	12.97 (4.76–35.35)	<0.01	7.68 (2.66–22.21)	<0.01
5	25.93 (10.95–61.40)	<0.01	7.16 (2.89–17.77)	<0.01	18.34 (10.82–31.08)	<0.01	6.98 (3.97–12.29)	<0.01	17.85 (6.64–48.00)	<0.01	8.77 (3.04–25.27)	<0.01
Path_T_stage(T3 or over)	5.40 (3.81–7.65)	<0.01	2.20 (1.47–3.30)	<0.01	3.65 (2.88–4.63)	<0.01	1.58 (1.20–2.08)	<0.01	3.76 (2.25–6.33)	<0.01	1.76 (0.97–3.19)	0.06
**Patho_N**												
N0	Reference		Reference		Reference		Reference		Reference		Reference	
N1	2.27 (1.44–3.56)	<0.01	1.33 (0.83–2.13)	0.23	2.29 (1.58–3.31)	<0.01		0.26	3.26 (1.31–8.08)	0.11		0.74
NX	0.33 (0.24–0.47)	<0.01	0.72 (0.49–1.04)	0.08	0.41 (0.32–0.52)	<0.01		0.29	0.53 (0.31–0.91)	0.20		0.28
Path_LVI (Positive)	4.46 (2.97–6.72)	<0.01		0.22	4.47 (3.33–6.01)	<0.01	1.60 (1.16–2.20)	<0.01	3.82 (2.12–6.89)	<0.01		0.71

## Discussion

This study aimed to evaluate differences in risk factors for BCR after radical prostatectomy stratified by the degree of obesity, focusing on the surgical methods used. Prostate cancer generally shows indolent course, and less than 10% of patients experience metastasis, progression, or death during short-term follow-up periods^16,17^. We used BCR as an oncological outcome in this study, which occurred in more than 30% of patients within 5 years follow-up^20^. The degree of obesity was not found to affect BCR-free survival. RALP showed better BCR-free survival than RRP. Statistically significant differences were found in the ‘overweight’ and ‘obese’ groups but not in the ‘normal’ group. After adjusting for confounders, the operation method did not affect BCR-free survival in the ‘normal’ group. In the ‘overweight’ and ‘obese’ groups, RALP showed gradual hazard reduction as the degree of obesity increased.

Obesity is one of the well-known risk factors for cancer-related mortality in many malignancies^3,22^. The relationship between obesity and prostate cancer mortality can be explained in various ways. Obesity is a surrogate marker of severity of metabolic syndrome. A recent meta-analysis^23^ revealed the negative correlation between metabolic syndrome and prostate cancer outcomes, including BCR. Obesity was associated with aggressive pathologic features by lowering the serum testosterone level or causing hyperinsulinemia^6^. Several studies have revealed that higher BMI is related to adverse pathologic factors and positive surgical margins^24^. Despite the available evidence, the relationship between obesity and oncological outcomes of prostate cancer remains controversial^5,6,8,9^. In this study, there was no direct association between obesity and BCR-free survival in the patients who underwent prostatectomy. This was likely because the study group consisted of patients who were treated with surgery alone; thus, obesity was found to have less impact on BCR than the pathologic or surgical factors, such as surgical margin or adverse pathologic features.

From the subgroup analysis, we showed that risk factors contributing to BCR differ between obesity subgroups. Interestingly, the survival benefit of RALP compared to that of RRP differed according to the degree of obesity. RALP was found to improve BCR-free survival in the ‘overweight’ and ‘obese’ groups with a more gradual decrease of HR than RRP. We anticipate that these results were caused by the compensating mechanism of robotic surgery for the surgical difficulty caused by obesity. The correlation between operation difficulty and obesity is well established. In addition to excessive fat in the pelvic cavity, a large prostate can also cause difficulty in visualizing the operation field during prostatectomy in obese men. As a result, prostatectomy in obese men increases wound complication^25^, intra-operative bleeding^26^, and operation time^27^. Robotic prostatectomy provides improved visualization and increases the accuracy of the procedure. Because RALP involves creating a pneumoperitoneum during the procedure, RALP is expected to overcome the operation difficulty problems in obese men during prostatectomy. As expected, we found that the RALP group showed lower apex and anterior margin positive rates than the RRP group (Table 2). Our study results provide useful information for patient counselling before prostatectomy and provide evidence regarding which patient groups benefit most from robotic surgery.

In this study, we used well-known risk factors for BCR for the adjustment of multivariable analysis^20,21^. The pre-operative PSA level, positive surgical margin, and Gleason grade group in the pathologic specimen were significant risk factors in all of the obesity subgroups. Preoperative PSA and the Gleason score after the operation are well-established risk factors for BCR after prostatectomy. The Cancer of the Prostate Risk Assessment (CAPRA)-S, Kattan, and the Duke prostate cancer (DPC) nomograms contained pre-operative PSA and pathologic Gleason score as a predictor of BCR after the operation^28^. Although not all margin-positive patients experience BCR, the presence of a positive surgical margin is a well-established risk factor for BCR^29^. The presence of lymphovascular invasion is also considered as a potential risk factor for BCR^30^. However, after adjusting for all confounding factors, only the ‘overweight’ group showed significant correlation between lymphovascular invasion and BCR. Considering that the presence of lymphovascular invasion indicates an increasing hazard of BCR in all study populations, this result could have been caused by a confounder effect or a small number of sub-group patients. After adjusting for confounders, the operation method used remained a contributing risk factor for BCR in the ‘overweight’ and ‘obese’ group.

This study has some limitations. Owing to its retrospective design, this study has an inevitable chance of selection bias. Another limitation is the size of the study population. Although we analysed the data of approximately 3000 patients, the ‘obese’ group contained only 378 patients. The definition of BMI and BCR varied compared to other studies, and thus, this difference was another limitation. Despite this limitation, we successfully analysed differences in the risk factors of BCR according to the degree of obesity. Finally, we found that RALP showed a gradual decrease in the HR of BCR in ‘overweight’ and ‘obese’ patients. Future well-matched, larger, prospective studies are warranted to compare RALP with RRP in obese patients to confirm our findings.

In summary, BMI was not directly associated with BCR-free survival in this study. After adjusting for confounding factors, robotic prostatectomy showed benefit on BCR-free survival over open prostatectomy in the ‘overweight’ and ‘obese’ groups. Moreover, gradual increment of hazard reduction may highlight this association. Well-designed studies are needed to assess the relationship between the degree of obesity and the benefit of robotic surgery in BCR-free survival.

## Data Availability

The datasets generated during and/or analysed during the current study are not publicly available due to included patient information but are available from the corresponding author on reasonable request.
